# Synthetic nicotine e-liquids sold in US online vape shops

**DOI:** 10.1016/j.pmedr.2023.102222

**Published:** 2023-04-26

**Authors:** Shaoying Ma, Zefeng Qiu, Jian Chen, Ce Shang

**Affiliations:** a Center for Tobacco Research, The Ohio State University Wexner Medical Center, Columbus, OH, USA; b Department of Computer Science and Engineering, The Ohio State University, Columbus, OH, USA; c Division of Medical Oncology, Department of Internal Medicine, The Ohio State University Wexner Medical Center, Columbus, OH, USA

**Keywords:** Synthetic nicotine, e-liquid, Vaping, Electronic cigarette, e-cigarette, Online vape shop, Electronic nicotine delivery systems, ENDS, Tobacco control

## Abstract

Synthetic nicotine (relative to tobacco-derived, or “natural” nicotine) is an emerging feature of e-cigarettes including e-liquids in the online marketplace. This study investigated a total of 11,161 unique nicotine e-liquids sold in online stores in the US during 2021, using keyword matching approach to identify the feature of synthetic nicotine based on product description texts. We showed that in 2021, 2.13% of nicotine-containing e-liquids in our sample were marketed as synthetic nicotine e-liquids. About a quarter of the synthetic nicotine e-liquids that we identified were salt-based; the nicotine strength varied; and those synthetic nicotine e-liquids had a variety of flavor profiles. Synthetic nicotine containing e-cigarettes are likely to remain in the market and manufacturers might market those products as “tobacco-free,” to attract consumers who this feature as healthier or less addictive. It is important to monitor synthetic nicotine in the e-cigarette marketplace and assess how this feature influences consumer behaviors.

## 1 Introduction

Synthetic nicotine (compared to tobacco-derived or “natural” nicotine) has emerged as a product feature of e-liquids for manufacturers to manipulate (Jordt, 2023, Duell et al., 2021, Davis et al., 2023). Two nicotine isomers (i.e., the geometry of the nicotine molecule) exist: (R)-nicotine and (S)-nicotine (Jordt, 2023, Ashley et al., 2009). Natural tobacco nicotine is over 99% (S)-nicotine (Jordt, 2023, Duell et al., 2021, Ashley et al., 2009). Initially, synthetic nicotine e-cigarettes, marketed as “tobacco-free”, contained a 50:50 mixture of S- and R-nicotine isomers, known as racemic nicotine (Jordt, 2023, Duell et al., 2021, Hellinghausen et al., 2017). However, manufacturers of synthetic nicotine e-cigarette products now offer both > 99% pure S-nicotine and a mixture of S- and R-nicotine isomers (Jordt, 2023, Cheetham et al., 2022). While the presence of isomer mixture indicates synthetic nicotine, >99% pure S-nicotine could be derived from either tobacco or synthetic sources (Jordt, 2023, Cheetham et al., 2022). Research has demonstrated that (R)-nicotine and (S)-nicotine have different pharmacologic potencies and effects in both animal and in vitro studies (Duell et al., 2021, Yang et al., 2019, Yildiz et al., 1998, Barlow and Hamilton, 1965). Therefore, e-cigarette products containing a mixture of S- and R-nicotine isomers may exhibit different properties and levels of addiction liability compared to those containing pure S-nicotine.

When the US Food and Drug Administration (FDA) issued marketing denial orders (MDOs) to a significant number of e-cigarette manufacturers in October 2021, the deeming rules were written in a way that considered nicotine products as “tobacco-derived” (U.S. Food Drug Administration, 2021). This definition left a loophole for e-cigarette companies (e.g., Puff Bar) to avoid FDA regulation and to continue to sell their products without a premarket tobacco product applications (PMTA) if they claimed to produce “synthetic” rather than “tobacco-derived” nicotine products (Duell et al., 2021, Majmundar et al., 2022).

## 2 Methods

This study investigates the prevalence of synthetic nicotine in 11,161 unique nicotine-containing e-liquid products sold across five national online vape shops in the US during 2021. We collected e-liquid data from these popular online stores using web scraping tools. To maintain the anonymity of the stores in our sample, we refer to them as stores 1–5 (Ma et al., 2022). Our selection process for the online vape shops consisted of the following steps: In January 2021, we conducted Google and Reddit searches to identify popular online vape shops in the US. We selected five stores as a convenience sample based on the search results. Our criteria included choosing the top three online stores without physical addresses from the Google search results and selecting two additional stores without physical addresses from the most recent Reddit discussion based on comments with the highest number of upvotes. As a result, stores 1–3 were from the top Google search results, while stores 4–5 were selected based on Reddit comments with the most upvotes. Notably, Store 2 appeared in both the top Google search results and Reddit user recommendations. Finally, we scraped web data from stores 1–5 between February and May 2021. The data in this study provides a snapshot of synthetic nicotine e-liquids available in popular US online vape shops.

**Fig. 1:**
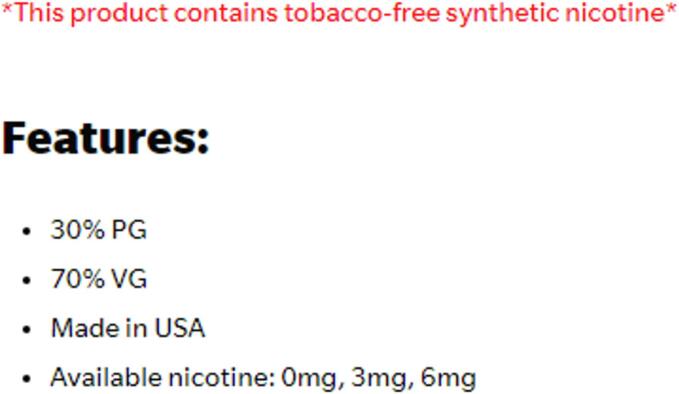
Example of product online description text showing e-liquid attributes and the claim of “tobacco-free synthetic nicotine”.

The current study uses product-level data that were scraped from store websites, which are publicly available and do not involve human subjects; thus, it is exempt from ethical compliance. We used keyword-matching techniques to identify synthetic nicotine e-liquids based on product description texts. Fig. 1 shows an example of product description text that mentions synthetic nicotine.

## 3 Results

We found that in 2021, 2.13% of nicotine-containing e-liquid products (238 out of 11,161 products) were marketed as containing synthetic nicotine. Among the 238 synthetic nicotine e-liquids we identified, 57 (23.95%) of them are salt-based; the nicotine strength varies from 3 mg/ml to 50 mg/ml, with a mean of around 14 mg/ml and a median of 6 mg/ml.

Those synthetic nicotine e-liquids have a variety of flavor profiles, e.g., fruit, menthol + tobacco, and alcohol + fruit + sweet; 216 (90.76%) of them contain at least one fruity flavor (i.e., berry, citrus, tropical, etc.); 171 (71.85%) contain sweet flavors (e.g., cookie, caramel, taffy); 100 (42.02%) contain tobacco flavors; 92 (38.66%) contain menthol/mint flavors; 22 (9.24%) contain nutty flavors (e.g., almond, peanut); 9 (3.78%) contain spice flavors (e.g., cinnamon, nutmeg); 10 (4.2%) contain coffee/tea flavors (e.g., mocha, espresso); 72 (30.25%) contain alcohol flavors (e.g., brew, punch, scotch, cooler); and 86 (36.13%) contain other beverage flavors (e.g., milk, lemonade, nectar, refresher, pop) (see Table 1).

**Table 1:** Flavor profiles seen in synthetic nicotine e-liquids.

**Flavor**	**n (%)** (N = 238)
Fruity (e.g., berry, citrus, tropical, etc.)	216 (90.76)
Sweet (e.g., cookie, caramel, taffy)	171 (71.85)
Tobacco	100 (42.02)
Menthol/Mint	92 (38.66)
Other Beverages (e.g., milk, lemonade, nectar, refresher, pop)	86 (36.13)
Alcohol (e.g., brew, punch, scotch, cooler)	72 (30.25)
Nutty (e.g., almond, peanut)	22 (9.24)
Coffee/Tea (e.g., mocha, espresso)	10 (4.2)
Spice (e.g., cinnamon, nutmeg)	9 (3.78)

## 4 Discussion

In recognition of this market trend, HR 2471, passed in 2022, granted the FDA regulatory authority over “any product made or derived from tobacco, or containing nicotine from any source, that is intended for human consumption,” which closed the loophole for manufacturers to exploit the “synthetic nicotine” claim (H.R., 2022, Stephenson, 2022). Nevertheless, the manufacturing and sales of e-cigarette products using synthetic nicotine are likely to remain because these products are now marketed as “tobacco-free,” a marketing strategy that could attract naïve consumers, particularly adolescents and young adults (AYAs), who might perceive “tobacco-free” products as healthier or less addictive (Chen-Sankey et al., 2021, Morean et al., 2022, Camenga et al., 2022).

With the nicotine features of e-cigarettes constantly evolving, the context of how other combustible and non-combustible products are regulated could further incentivize or disincentivize e-cigarette use. While the enforcement of PMTA and MDO will remove a large number of popular e-cigarette options, including popular nicotine options, the FDA also announced that it intends to establish a maximum level of nicotine in cigarettes and certain other combustible tobacco–a move that could incentivize combustible tobacco smokers to switch to e-cigarettes that can deliver nicotine concentrations at a level comparable to cigarettes (U.S. Food & Drug Administration, 2022). Monitoring nicotine features (concentration, form, and isomer) in the marketplace and assessing how these features influence consumer behaviors are crucial for the FDA to design effective e-cigarette nicotine standards that balance population health impacts.

## Funding

This study was funded by The Ohio State University Comprehensive Cancer Center (OSUCCC) Center for Tobacco Research Pilot Grant (PI: Ce Shang; 1/1/2021–06/30/2021). Dr. Ce Shang is funded by the National Cancer Institute (R21CA249757; PI: Ce Shang; 9/16/2021–8/30/2023). Dr. Shaoying Ma is funded by the Pelotonia Fellowship Program at OSUCCC.

## CRediT authorship contribution statement

**Shaoying Ma:** Methodology, Software, Validation, Formal analysis, Investigation, Data curation, Writing – original draft, Writing – review & editing, Visualization, Project administration. **Zefeng Qiu:** Methodology, Software, Validation, Formal analysis, Investigation, Data curation, Visualization, Project administration. **Jian Chen:** Methodology, Validation, Resources, Supervision, Funding acquisition. **Ce Shang:** Conceptualization, Methodology, Validation, Investigation, Resources, Writing – original draft, Writing – review & editing, Visualization, Supervision, Funding acquisition.

## Declaration of Competing Interest

The authors declare that they have no known competing financial interests or personal relationships that could have appeared to influence the work reported in this paper.

## Data Availability

Data will be made available on request.
